# Rapid formation and evolution of an extreme haze episode in Northern China during winter 2015

**DOI:** 10.1038/srep27151

**Published:** 2016-05-31

**Authors:** Yele Sun, Chen Chen, Yingjie Zhang, Weiqi Xu, Libo Zhou, Xueling Cheng, Haitao Zheng, Dongsheng Ji, Jie Li, Xiao Tang, Pingqing Fu, Zifa Wang

**Affiliations:** 1 State Key Laboratory of Atmospheric Boundary Layer Physics and Atmospheric Chemistry, Institute of Atmospheric Physics, Chinese Academy of Sciences, Beijing 100029, China; 2 Center for Excellence in Urban Atmospheric Environment, Institute of Urban Environment, Chinese Academy of Sciences, Xiamen 361021, China; 3 College of Applied Meteorology, Nanjing University of Information Science and Technology, Nanjing 210044, China; 4 School of Atmospheric Physics, Nanjing University of Information Science and Technology, Nanjing 210044, China; 5 University of Chinese Academy of Sciences, Beijing 100049, China; 6 Key Laboratory of Environmental Optics & Technology, Anhui Institute of Optics and Fine Mechanics, Chinese Academy of Sciences, Hefei 230031, China

## Abstract

We investigate the rapid formation and evolutionary mechanisms of an extremely severe and persistent haze episode that occurred in northern China during winter 2015 using comprehensive ground and vertical measurements, along with receptor and dispersion model analysis. Our results indicate that the life cycle of a severe winter haze episode typically consists of four stages: (1) rapid formation initiated by sudden changes in meteorological parameters and synchronous increases in most aerosol species, (2) persistent evolution with relatively constant variations in secondary inorganic aerosols and secondary organic aerosols, (3) further evolution associated with fog processing and significantly enhanced sulfate levels, and (4) clearing due to dry, cold north-northwesterly winds. Aerosol composition showed substantial changes during the formation and evolution of the haze episode but was generally dominated by regional secondary aerosols (53–67%). Our results demonstrate the important role of regional transport, largely from the southwest but also from the east, and of coal combustion emissions for winter haze formation in Beijing. Also, we observed an important downward mixing pathway during the severe haze in 2015 that can lead to rapid increases in certain aerosol species.

Northern China experiences frequent severe haze episodes, particularly in winter^1^,^2^,^3^,^4^,^5^. High concentrations of fine particulates (PM_2.5_) not only cause substantially reduced visibility by scattering and absorbing sunlight^6^, but also have detrimental effects on human health^7^. To address this issue, the Chinese State Council released the “Atmospheric Pollution Prevention and Control Action Plan” on September 10, 2013, which aims to reduce PM_2.5_ in the Jing-Jin-Ji area by 25% by 2017^8^. The National Development and Reform Commission along with the Ministry of Environmental Protection also released the “Coordinated Development of Ecological Environment Protection Plan in Jing-Jin-Ji Area” on December 30, 2015^9^. The annual mean PM_2.5_ concentration in Beijing decreased from 89.5 μg m^−3^ in 2013 to 80.6 μg m^−3^ in 2015; however reaching the ambitious goal of 64 μg m^−3^ by 2020 in the Jing-Jin-Ji area remains a challenge due to the complex sources (e.g., local emissions versus regional transport) and evolutionary mechanisms of PM_2.5_ under stagnant meteorological conditions.

Extensive studies have been conducted in recent years to investigate the sources and formation mechanisms of severe haze episodes in northern China^1^,^2^,^3^,^4^,^5^,^10^,^11^. The results indicate that the formation of severe haze episodes is tightly associated with stagnant meteorological conditions that are generally characterized by temperature (*T*) inversions, low wind speed (WS) and high relative humidity (RH)^1^,^10^,^11^,^12^. The formation of haze episodes is often rapid, with PM_2.5_ concentrations increasing from a few μg m^−3^ to tens and even hundreds of μg m^−3^ in less than a day^1^,^3^,^11^. Chemical composition analysis shows that secondary inorganic aerosols (SIA = sulfate + nitrate + ammonium) are dominant in aerosol particles, pointing to the key role of regional transport in the formation of severe haze episodes^1^,^11^,^13^,^14^. For example, it was estimated that regional transport contributed 53–100% of different aerosol species during the severe haze episode on January 12–13, 2013^1^. Several studies have also found a faster increase in sulfate than in nitrate during the evolution of haze, and that this is strongly associated with RH and high concentrations of NO_2_^1^,^11^,^15^,^16^. These results indicate that aqueous-phase processing at high RH levels affects the evolution of the chemical properties of haze during episodes. Source analysis further highlights the dominance of SIA in particulate matter (PM)^1^,^4^,^17^, and at times, secondary organic aerosols (SOA) make comparable contributions to SIA^18^. But, results also indicate that there is a considerable contribution of primary OA (POA), particularly coal combustion OA (CCOA), to PM during winter^5^,^19^. Together, these results demonstrate the complexity of the sources, formation mechanisms, and evolution of severe haze episodes.

Despite substantial efforts to reduce anthropogenic emissions during the last decade, Beijing still experiences frequent severe haze pollution^1^,^10^,^20^,^21^. The reasons for the rapid formation and evolution of such severe haze episodes, however, are still not well understood. In this study, we characterize a severe haze episode that occurred during November 26–December 1, 2015 using comprehensive ground and vertical measurements that include aerosol composition, gaseous species, optical properties, meteorological parameters, and turbulence data, along with model analysis using positive matrix factorization (PMF) and FLEXPART. We demonstrate the typical life cycle of a severe winter haze episode and its relationship with meteorological parameters, and elucidate its sources, rapid formation and evolutionary mechanisms.

**PM_2.5_ Mass Concentrations and Meteorological Conditions** The severe haze episode occurred between November 26 and December 1, 2015, and persisted for more than five days (Fig. 1). The PM_2.5_ mass exhibited a nonlinear increase during the 5-day evolution period with an hourly maximum concentration of 626 μg m^−3^ on November 30, similar to that observed during the severe haze episode in January 2013 (680 μg m^−3^)^10^. The PM_2.5_ concentration was more than 8 times the Chinese National Ambient Air Quality Standard (24-hour mean of 75 μg m^−3^) and 25 times that of the World Health Organization standard (24-hour mean of 25 μg m^−3^). This PM_2.5_ concentration was the highest value recorded in 2015 and led to the first “orange” air quality alert (i.e., severe air pollution lasting for 3 days^22^) ever issued in Beijing.

Meteorological conditions were stagnant during the entire pollution period. As shown in Fig. 1, WS was consistently low, less than 2 m s^−1^ below 400 m (Fig. 1c), while RH continuously increased to high levels (near 100%), and remained over 60% for most of the five day period. Water vapor levels increased along with RH, from 3.2 to 5.8 g m^−3^, and aerosol liquid water content also increased, with values above 0.05 g m^−3^ during the final day (Ep5, Fig. 1e), which indicated the presence of a severe fog event. The turbulence kinetic energy (TKE) in the horizontal (*u* and *v*) and vertical (*w*) wind components decreased significantly at heights below 300 m and remained at consistently low values (<0.5 m^2^ s^−2^) during the haze episode. The friction velocity (u_*_) decreased in a similar way to TKE, exhibiting values less than 0.4 m s^−1^ (Fig. S1). These results together suggest that there were stagnant conditions during the entire haze period, which was likely the primary external factor driving the formation of the severe haze episode.

The evolution of this severe haze episode was more complex than those previously reported in Beijing^1^,^3^,^4^,^23^,^24^,^25^ because it persisted longer, had multiple incidences of rapid aerosol increases (e.g., F1–F4 in Fig. 1), exhibited downward mixing of air pollutants (e.g., D1–D4 in Fig. 1), involved fog processing (Ep5), and had several temporary aerosol reductions due to meteorological changes. The PM_2.5_ formation rates varied widely among the four formation stages with the highest rates of increase in F1 and F3 (115 and 165 μg m^−3^ h^−1^, respectively). The formation rates were much higher than those observed during severe haze episodes in October 2014 (2–4.4 μg m^−3^ h^−1^)^3^, and were also higher than during the January 2013 haze episode (88 μg m^−3^ h^−1^)^10^. The different formation rates and evolution processes can be mainly attributed to differences in meteorological conditions, as the primary source emissions and secondary aerosol formation mechanisms were similar for the 2015 haze episode and previous ones. For instance, the winds below 400 m were largely from the north and east except during the formation episode (Ep2), which had a prevailing southwesterly wind (Fig. 1). This is different from severe haze episodes previously observed in Beijing, including those during the same period in 2014 (Fig. S2), that were typically related to south-southwesterly winds through the entire vertical layer^25^. Moreover, WS was low (<2 m s^−1^) through the entire vertical layer below 400 m during the 2015 haze episode (Fig. 1d), while large vertical gradients were observed during the 2014 haze episodes.

**Discussion of the Formation and Evolution of the 2015 Severe Haze Episode** We demonstrate that the chemical evolution of this severe haze episode consisted of four stages: (1) a formation stage (Ep2), (2) an evolution stage (Ep3), (3) further evolution associated with fog events (Ep5), and (4) a clearing stage (Ep6) (Fig. 2). Ep4 is an exception to this evolutionary pattern as it exhibited strong influences from local sources. The formation of each of the four episodes (F1–F4 in Fig. 1) was initiated by a sudden change in meteorological parameters that was characterized by an increase in RH, a decrease in *T*, and a change in wind direction (WD). This indicates that the formation of severe haze episodes is primarily driven by meteorological changes. While the mean PM_2.5_ mass concentration increased gradually from 131 μg m^−3^ (Ep2) to 462 μg m^−3^ (Ep5), aerosol chemical composition exhibited substantial changes. Secondary aerosols (=SIA + SOA) that are principally formed on a regional scale dominated particle composition during three episodes (Ep2, Ep3, and Ep5), on average accounting for 67% of the total PM_1_ mass, while the contributions of POA varied from 20% to 23%. These results highlight the important role of regional transport in the formation of severe haze episodes. Indeed, all aerosol species were observed to have synchronous increases during the formation stages of haze episodes, supporting the explanation that regional transport is important.

Secondary and primary aerosol species evolved differently during the severe haze episode. As indicated in Fig. 2, all secondary aerosol species evolved similarly and remained at relatively constant concentration levels during the entire period. In contrast, the variations in primary aerosol species were more dynamic, with enhanced concentrations in early morning due to coal combustion emissions and at noon and evening meal times due to cooking emissions. Specifically, CCOA was the dominant contributor to OA, accounting for 33–37% except during Ep2 (15%). These results suggest that coal combustion was the dominant primary emission source in winter in Beijing, consistent with the conclusions from previous studies^1^,^18^,^19^,^26^. During the evolution stage, the composition of secondary aerosols changed, with an increase in sulfate from 10% to 21%, yet the changes in nitrate were not significant, from 13–16%. As a result, the average ratio of SO_4_/NO_3_ increased from 0.66 (Ep2) to 1.54 (Ep5) associated with a decrease of SO_2_/NO_2_ ratios from 0.40 to 0.18. These results clearly indicate the enhanced role of aqueous-phase production of sulfate during the evolution stage. The aqueous-phase production of sulfate was strongly associated with aerosol liquid water content^1^,^27^, which increased significantly from 3.6 mg m^−3^ (Ep2) to 150 mg m^−3^ (Ep5, a severe fog event). Therefore, the increased sulfate contribution was mainly caused by aqueous-phase production via fog processing of SO_2_^27^, particularly under high NO_2_ conditions^15^,^16^. This is also consistent with the high sulfur oxidation ratio (~0.4, molar ratio of sulfur in sulfate to the total sulfur) during this episode. In comparison, the mixing ratio of NO_2_ was relatively constant during the entire episode (Fig. S3) indicating that aqueous-phase oxidation of NO_2_ was unlikely to be important. One explanation for this is that the extremely low O_3_ inhibited the formation of nitrate radicals and dinitrogen pentoxide (N_2_O_5_), two nitrogen oxides with dominant sinks involving heterogeneous reactions^28^. The correlations between sulfate and nitrate further demonstrate their disparate formation mechanisms at different RH levels. As shown in Fig. S4, sulfate was correlated with nitrate at both low and high RH levels (r^2^ = 0.96–0.99), and the regression ratios were also similar (1.09 at high RH levels, 1.04 at low RH levels). However, the intercept of 13.9 μg m^−3^ indicates additional production of sulfate at high RH levels, which contributed 30–45% of the total sulfate during the three episodes. This result confirms that aqueous-phase production at high RH levels played an important role in sulfate evolution during the severe haze episode^27^. The changes in aerosol composition also led to a change in optical properties. For example, the mass extinction efficiency of PM_2.5_ (=*b*_ext_/PM_2.5_) increased from 4.1 (Ep2) to 5.8 m^2^ g^−1^ (Ep5), and this was associated with an increase in single scattering albedo (SSA) from 0.90 to 0.94 (Fig. S3).

The evolution of the severe haze episode was temporarily interrupted in Ep4 which was characterized by an enhanced contribution of POA (35%) and a decrease in secondary aerosols (53%). This indicates that Ep4 was strongly influenced by local source emissions. The OA composition during Ep4 consistently showed an increase in local cooking aerosols, from 8–9% to 32%, and the total POA increased from 42–49% to 65%. We also observed very different vertical profiles of meteorological parameters during Ep4 compared to the other three episodes. During Ep4, *T* showed a strong inversion below 140 m (Fig. 3a), and RH and WS both exhibited strong vertical gradients with higher RH (70–80%) and lower WS (~1 m s^−1^) at ground level compared to higher altitudes (~50% and 3 m s^−1^, respectively, Fig. 3b,c). Such vertical structure in meteorological parameters would create a stagnant layer near ground level, leading to rapid increases in local pollutants. Indeed, gaseous NO and CO increased with organic aerosols from cooking during this episode (Fig. S3). The presence of high concentrations of secondary aerosol species (e.g., SOA, SO_4_, and NO_3_), on the other hand, suggests that this was mixed with regional transport and local source emissions.

Figure 4 shows the footprint regions and spatial distributions of PM_2.5_ for the six episodes in Fig. 1. The early formation of the first major episode (Ep2) was associated with a change in air mass and wind direction from northerly to west-southwesterly. Although the footprint region of Ep2 near ground level (50 m, Fig. 4b) is to the northwest, we observed a clear footprint region to the southwest at higher altitudes (500 m, Fig. S5), which is consistent with the prevailing southwesterly winds (Fig. 1a). The vertical profiles show relatively high wind speeds (2–4 m s^−1^, Fig. 1) between 50 m–600 m, clearly indicating regional transport from the southwest. Indeed, a long, narrow region southwest of Beijing with high PM_2.5_ concentrations was formed, and the spatial distribution of the surface wind field suggests regional transport from the southwest to the northeast (Fig. 4h). The footprint region of Ep3 near ground level was mainly located to the north and east, while it was dominantly to the east at 500 m. By comparing with the spatial distribution of PM_2.5_ (Fig. 4i), we infer that the formation of Ep3 was likely due to the recirculation of air pollution from the east. Ep5 was characterized by a clear footprint region located to the southwest (mainly over Hebei province) and east, indicating that it was a mix of eastern and southwestern air masses which led to the formation of the most severe haze episode (Ep5) in 2015. The above results demonstrate two major transport pathways, from the southwest and from the east, in the formation of this severe winter haze episode. It is interesting to note that the spatial distribution of PM_2.5_ concentrations in northern China exhibits a clear east-west division along the Taihang Mountains, and a north-south division near the center of Beijing (Fig. 5h–l). As a result, clean and polluted air masses can alternate at our sampling site depending on the dominant wind fields, and this may lead to rapid increases and decreases in aerosol species during the evolution of severe haze episodes (Figs 1 and 2). Combining the conclusions from footprint and compositional analyses, our results have the significant implication that mitigating severe winter haze in northern China requires a priority on control of secondary aerosol precursors and coal combustion emissions on a regional scale.

During the evolution of this severe episode, we observed multiple air pollution events (D1–D4 in Figs 1 and 2) that were likely a result of the downward mixing of southwesterly air masses from high altitudes to ground level and led to rapid increases in certain aerosol species. For instance, events D3 and D4 both showed rapid increases in PM_2.5_ concentration from ~400 μg m^−3^ to 600 μg m^−3^ in just one hour, returning to the previous levels in the following hour. We calculated the differences in aerosol chemical composition that were caused by the additional air mixing downwards. Sulfate, organics, and chloride showed significant increases during the two events, while changes in nitrate were insignificant (Fig. S6c). This is similar to the composition reported in Wang *et al.*^29^ who showed that coal combustion emissions had a dominant impact on these species. In fact, the difference in OA composition was dominated by CCOA, accounting for 45% and 53% during D3 and D4, respectively (Fig. S6b). These results suggest the presence of coal combustion-related air masses at high altitudes over Beijing, which can deteriorate the air quality of Beijing substantially when mixed downwards.

The severe haze episode was cleared at 22:10 on December 1 by strong, dry westerly winds. All aerosol species were reduced to extremely low levels (less than 2 μg m^−3^ in one hour), and the total PM_1_ mass decreased from ~150 μg m^−3^ to less than 4 μg m^−3^. It is interesting to note that the PM_1_ composition of the subsequent clean episode (Ep6) was substantially different from that of Ep1 (Fig. 2). Ep6 had a much higher sulfate contribution than Ep1 (35% vs. 15%). A possible explanation for this is that aerosol particles during Ep6 consisted of residual particles from the evaporation of fog droplets formed in Ep5. The differences in composition also led to a different SSA, which was 0.91 for Ep6 and 0.86 for Ep1. Similar compositional differences between clean periods were also observed in 2014 (Ep1 vs. Ep7 in Fig. S2) and January 2013^1^.

**Comparisons with Severe Haze Episodes in Winter 2014** The formation and evolution of the 2015 severe haze episode was compared with those observed during the same winter period in 2014 (see supplementary for details). The formation of the two haze episodes in 2014 (F1 and F2 in Fig. S2) was also initiated by a change in wind direction bringing air from the south, an increase in RH and a decrease in *T*. However, the dominant southerly and southwesterly winds through the entire vertical layer (Fig. S2a) led to a much slower accumulation of secondary aerosol species. The evolution of 2014 haze episodes was slightly different from that in 2015, and was influenced by clear mountain-valley breezes (e.g., M1 and M2 in Fig. S2), but ended similarly with fog events associated with high liquid water content and significantly enhanced sulfate concentrations. As indicated in Fig. 5a,b, the correlations (r^2^ = 0.91–0.99) and ratios of combustion tracers (e.g., BC, CO, and NO_x_) were similar during the haze episodes in 2014 and 2015, and were 2.69 and 2.58, respectively for BC/CO, and 27.4 and 25.3, respectively for NO_x_/CO. POA from traffic, coal combustion, and cooking sources was consistently and tightly correlated with BC (r^2^ = 0.79 and 0.98 for 2015 and 2014, respectively) and the slopes were also similar (2.98 vs. 2.87). Together this indicates similar primary emission sources for the two years. A higher POA/BC ratio was observed in Ep4 during 2015, mainly due to enhanced cooking emissions with a much smaller emission ratio for BC^30^. Figure 5e shows a strong, positive correlation between NO_2_ and NO_3_ (r^2^ = 0.91–0.98), along with similar NO_2_/NO_3_ ratios in 2014 and 2015 (1.95 and 2.25, respectively). This result further indicates that oxidation of NO_2_ to form NO_3_ was not significant during the severe haze episodes. In fact, the NO_2_/NO_x_ ratio was relatively constant among different episodes (0.30–0.32, Fig. 5c), and variations in nitrate and NO_2_ concentrations were small (Fig. S7). In addition, we observed similar correlations between SOA and nitrate in 2014 and 2015 (Fig. 5f). SOA and nitrate were tightly correlated in both 2014 and 2015 (r^2^ = 0.93–1.0), and the SOA/NO_3_ ratios were also similar (1.14 vs. 1.22). This result demonstrates that secondary formation mechanisms were similar in 2014 and 2015. Thus, we conclude that differences in the evolution of the severe haze episodes in 2014 and 2015 were largely due to the different meteorological conditions.

## Methods

### Instrumentation and Measurements

All measurements were made at an urban site in Beijing located at the Institute of Atmospheric Physics (IAP, 39°58′N, 116°22′E; elevation: 49 m ASL) from November 25 to December 2, 2015. All instruments were stored in a room on the roof of a two-story building and the sampling height was approximately 8 m above ground level. The non-refractory submicron aerosol (NR-PM_1_) species, including organics (Org), sulfate (SO_4_), nitrate (NO_3_), ammonium (NH_4_) and chloride (Cl) were measured with an Aerodyne Aerosol Chemical Speciation Monitor (ACSM). The setup and operation of the ACSM followed the protocols in Sun *et al.*^23^. Black carbon (BC, 880 nm) was measured with a seven-wavelength Aethalometer (AE33, Magee Scientific Corp.). The absorption coefficient (*b*_ap_) at 630 nm was calculated using a constant mass absorption efficiency of 7.3 m^2^ g^−1^ 31, i.e., *b*_ap_ = [BC] × 7.3. The light extinction (*b*_ext_, λ = 630 nm) of dry fine particles (PM_2.5_) was measured with a Cavity Attenuated Phase Shift (CAPS) Extinction Monitor with a 1 s time resolution^32^. The SSA at 630 nm was then calculated as SSA = (*b*_ext_ − *b*_ap_)/*b*_ext_. The gaseous species, including CO (Model 48*i*), NO/NO_y_ (Model 42*i*), O_3_ (Model 49*i*), and SO_2_ (Model 43*i*) were measured simultaneously at the same site with gas analyzers from Thermo Scientific.

Meteorological parameters including WS, WD, RH, and *T*, were measured at 15 heights (8, 15, 32, 47, 63, 80, 100, 120, 140, 160, 180, 200, 240, 280, and 320 m) on the Beijing 325 m meteorological tower. The vertical profiles of three wind components (*u*, *v*, *w*) between 100 m and 6000 m were measured by a Doppler wind lidar (Windcube 200, Leosphere, Orsay, France) at the same location. Turbulent wind velocity, virtual temperature, and pressure were measured with ultrasonic anemometers (Gill Instruments Limited, Lymington, UK), and the mixing ratios of water vapor (H_2_O) and carbon dioxide (CO_2_) were measured with Li-7500CO_2_/H_2_O gas analyzers (LI-COR, Inc., Nebraska, USA) at 7 heights (8, 15, 47, 80, 140, 200, and 280 m) on the tower. The friction velocity (u_*_) and the TKE of the three wind components (*u*, *v*, *w*) were then calculated (see supplementary for details). All data in this study are reported using Beijing Standard Time.

During the same winter period of 2014, an Aerodyne High-Resolution Time-of-Flight Aerosol Mass Spectrometer (AMS hereafter) was deployed in a ground trailer (the sampling height is approximately 4 m) at the same location. The size-resolved NR-PM_1_ species were measured at a time resolution of 2 min from November 24 to December 1, 2014. The setup, calibration, and operation of the AMS is detailed in Xu *et al.*^2^. Collocated measurements included BC with a two-wavelength Aethalometer (AE22, Magee Scientific Corp.), fine particle extinction coefficient with a CAPS PM_ext_ monitor, and gaseous species (CO, O_3_, NO/NO_2_, and SO_2_) with a range of gas analyzers from Thermo Scientific.

### ACSM and AMS Analyses

The ACSM and AMS data were analyzed with standard data analysis software^33^. Similar to our previous winter studies^19^,^23^, a constant collection efficiency (CE) of 0.5, commonly used in field campaigns^34^, was applied to both the HR-AMS and the ACSM datasets to determine the mass concentrations of NR-PM_1_ species. The choice of CE = 0.5 is reasonable because RH, particle acidity and the mass fraction of ammonium nitrate do not affect CE substantially^35^.

The organic aerosol spectra matrices of the ACSM and the AMS were then analyzed by PMF^36^ to resolve the potential OA factors from different sources and processes. Detailed procedures for data pre-treatment and error matrices for the PMF analysis are given in Ulbrich *et al.*^37^. The PMF results were evaluated following the steps recommended in Zhang *et al.*^38^ with a PMF diagnostics tool written in IGOR Pro^37^. After careful evaluation of the PMF results, a five-factor solution that included hydrocarbon-like OA (HOA), CCOA, cooking OA (COA), semi-volatile oxygenated OA (SV-OOA), and low-volatility OOA (LV-OOA) was chosen for both the ACSM and the AMS dataset. The mass spectral profiles and time series of five OA factors resolved in 2014 and 2015 are shown in Figs S8 and S9. The mass spectrum of LV-OOA was characterized by high *m/z* 44, and the time series was strongly correlated with SIA (r^2^ = 0.81 and 0.85, respectively). SV-OOA with high *m/z* 43/44 ratios was correlated with nitrate in both 2014 (r^2^ = 0.83) and 2015 (r^2^ = 0.76). The three POA factors exhibited very different mass spectral profiles and time trends. While the mass spectrum of COA was characterized by high *m/z* 55/57, typical characteristics of cooking emissions^39^, those of HOA and CCOA were characterized by prominent hydrocarbon ion peaks C_n_H_2n−1_^+^ and C_n_H_2n+1_^+^. The time series of COA had pronounced peaks that occurred at noon and evening meal times, whereas those of HOA and CCOA were generally correlated with the tracers for combustion emissions, including BC, CO and NO_x_ (r^2^ > 0.56). Because previous studies have found that a considerable fraction of BC is associated with secondary aerosols from regional transport^40^, a linear regression analysis of BC with the five OA factors was performed to determine the local and regional BC in this study^26^. The BC associated with HOA, COA, and CCOA was defined as local primary BC (BC_pri_) and that associated with SV-OOA and LV-OOA was defined as regional BC (BC_sec_).

### FLEXPART Analysis

A footprint region indicating the air mass origin for each episode was determined using two-day backward simulations of the Lagrangian particle dispersion model FLEXPART^41^ driven by meteorological fields (spatial resolution = 10 km, time resolution = 1 hour). Meteorological simulations were carried out using the Weather Research and Forecast model version 3.4 (WRF)^42^ with the National Centers for Environmental Prediction (NCEP) global reanalysis data as the initial and boundary conditions. Particle locations were then calculated with WRF-FLEXPART^43^. In the simulations, 10,000 tracer particles were released from the observation site at two heights, 50 m and 500 m, respectively, and the model was run backwards in time to determine the source areas and transport pathways of air pollutants during the specified period. A larger number of tracers in a cell indicates a greater impact from surface emission sources. A more detailed description of WRF-FLEXPART is given in supplementary.

### PM_2.5_ Simulations

The Nested Air Quality Predicting Modeling System (NAQPMS)^44^ developed by IAP, Chinese Academy of Sciences was used to simulate the PM_2.5_ concentrations in northern China. NAQPMS couples real-time emissions, advection, diffusion, dry and wet deposition, aerosol, and gaseous, aqueous phase, and heterogeneous chemical reactions, and also incorporates tracer-tagging and data assimilation techniques and a two-way nested module to consider interactions between the boundary layer and air pollution. A more detailed description of the NAQPMS model is given elsewhere^13^. As indicated in Fig. S13, the PM_2.5_ concentrations simulated with NAQPMS agree reasonably well with observations at different sites in northern China.

### Liquid Water Content

ISORROPIA-II^45^,^46^ was used to predict liquid water content associated with inorganic species. The aerosol composition measured with the ACSM and HR-AMS, and the meteorological conditions at the height of 8 m were input into the ISORROPIA-II model. ISORROPIA-II then calculates the composition and phase state of a K^+^-Ca^2+^-Mg^2+^-NH_4_^+^-Na^+^-SO_4_^2−^-NO_3_^−^-Cl^−^-H_2_O in thermodynamic equilibrium with gas-phase precursors.

## Additional Information

**How to cite this article**: Sun, Y. *et al.* Rapid formation and evolution of an extreme haze episode in Northern China during winter 2015. *Sci. Rep.* **6**, 27151; doi: 10.1038/srep27151 (2016).

## Supplementary Material

Supplementary Information

## Figures and Tables

**Figure 1:**
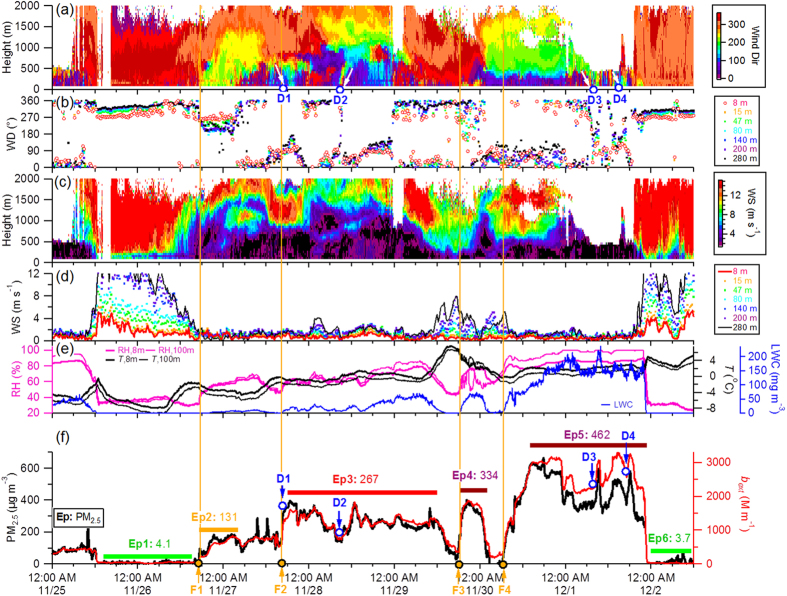
Evolution of (**a,b**) wind direction (WD), (**c,d**) wind speed (WS), (**e**) relative humidity (RH), temperature (*T*) and liquid water content (LWC), and (**f**) PM_2.5_ mass concentration and particle extinction coefficient (*b*_ext_) from November 25 to December 2, 2015. Six episodes (Ep1–Ep6) are marked for further discussion. Four initial stages in the formation of the episodes (F1–F4) and four events with evident downward mixing (D1–D4) are also marked.

**Figure 2:**
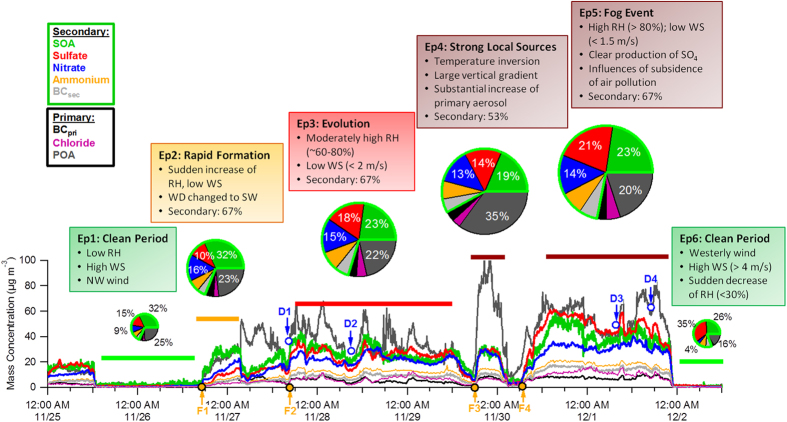
Evolution of aerosol chemical composition during the severe haze episode in 2015. Pie charts show the average chemical composition for each episode and the description of each episode is also included in the figure.

**Figure 3:**
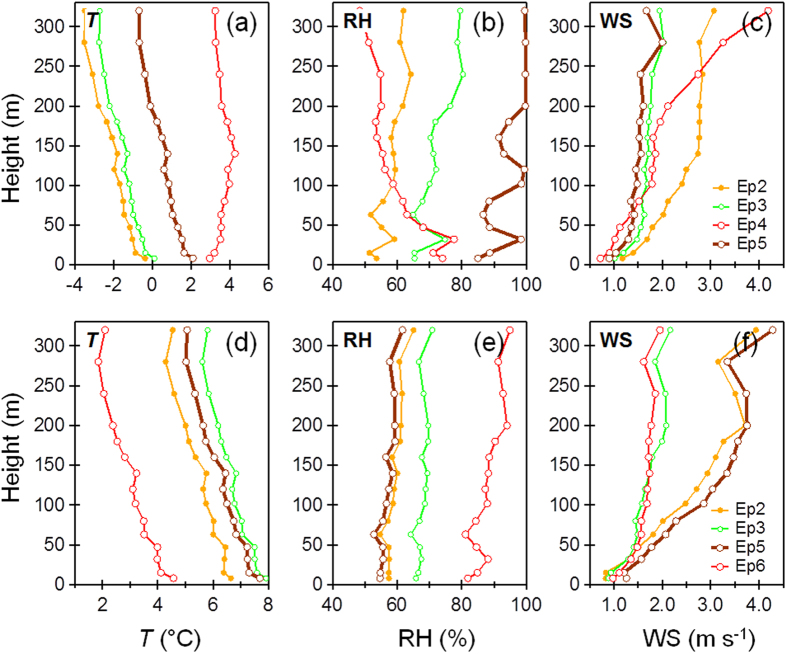
Average vertical profiles of meteorological parameters during four haze episodes in 2015 (**a–c**) and 2014 (**d–f**). The temperature of Ep2 in 2015 was offset by +3 °C for clarity.

**Figure 4:**
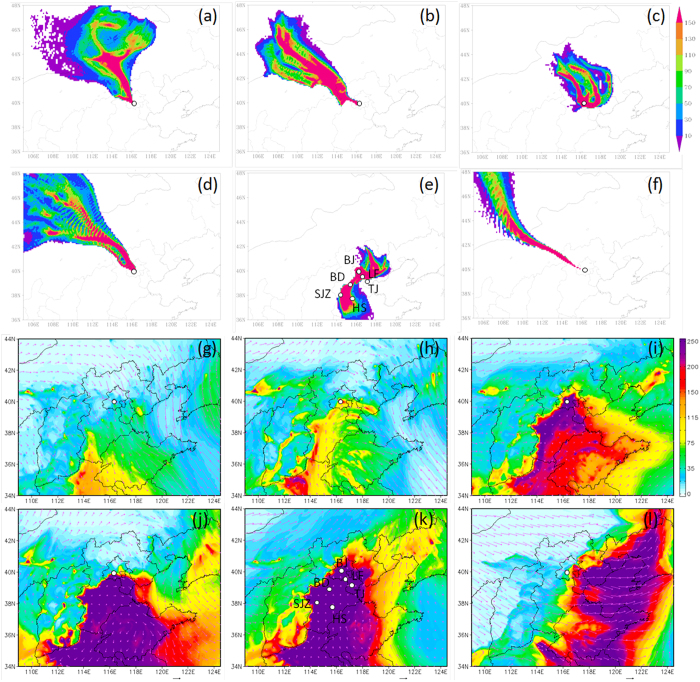
(**a–f**) Footprint regions of air mass origin for air arriving at 50 m altitude for the six episodes (Ep1–Ep6) marked in Figs 1 and 2. The legend indicates the concentrations of tracer particles. (**g,h**) Simulated average spatial distributions of PM_2.5_ (μg m^−3^) and surface wind fields (m s^−1^) for the six episodes (Ep1–Ep6). The cities marked in (**e**,**k**) are Beijing (BJ), Tianjin (TJ), Langfang (LF), Baoding (BD), Shijiazhuang (SJZ), and Hengshui (HS). The maps were drawn by IGOR Pro (version 6.3.7.2, WaveMetrics, Inc., Oregon USA), http://www.wavemetrics.com/.

**Figure 5:**
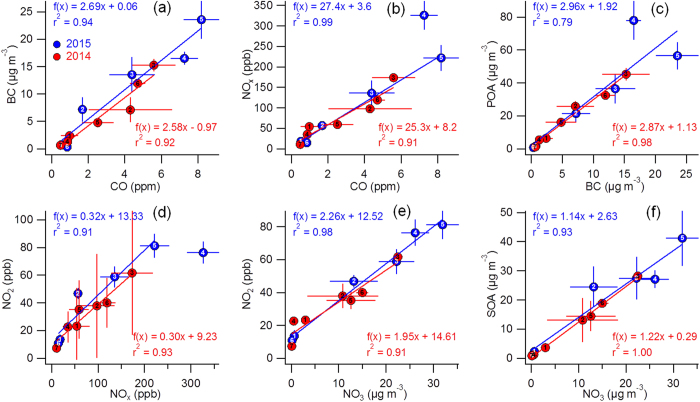
Scatter plots of (**a**) BC vs. CO, (**b**) NO_x_ vs. CO, (**c**) POA vs. BC, (**d**) NO_2_ vs. NO_x_, (**e**) NO_2_ vs. NO_3_, and (**f**) SOA vs. NO_3_ for the episodes marked in Fig. 2 in 2015 (blue) and Fig. 3 in 2014 (red). Error bars show one standard deviation. Note that Ep4 was excluded from the 2015 linear fit in (**b,d**) due to strong local source influences.
