# Supplementary material for: Rapid formation and evolution of an extreme haze episode in Northern China during winter 2015
Source: Sci Rep. 2016 May 31;6:27151. doi: 10.1038/srep27151 (PMC4886685; doi:10.1038/srep27151)
Supplement: Supplementary Information [file srep27151-s1.pdf]

Supplementary Information

Rapid formation and evolution of an extreme haze episode in Northern China during winter 2015

Yele Sun[1,2*], Chen Chen[1,4], Yingjie Zhang[1,5], Weiqi Xu[1,3], Libo Zhou[1], Xueling Cheng[1], Haitao Zheng[1,6], Dongsheng Ji[1], Jie Li[1], Xiao Tang[1], Pingqing Fu[1], Zifa Wang[1]

[1]State Key Laboratory of Atmospheric Boundary Layer Physics and Atmospheric Chemistry, Institute of Atmospheric Physics, Chinese Academy of Sciences, Beijing 100029, China

[2]Center for Excellence in Urban Atmospheric Environment, Institute of Urban Environment, Chinese Academy of Sciences, Xiamen 361021, China

[3]University of Chinese Academy of Sciences, Beijing 100049, China

[4]College of Applied Meteorology, Nanjing University of Information Science and Technology, Nanjing 210044, China

[5]School of Atmospheric Physics, Nanjing University of Information Science and Technology, Nanjing 210044, China

[6]Key Laboratory of Environmental Optics & Technology, Anhui Institute of Optics and Fine Mechanics, Chinese Academy of Sciences, Hefei 230031, China

*Corresponding author email: sunyele@mail.iap.ac.cn; Phone: +86-10-8202-1255

Severe Haze Episodes in 2014.

Two severe haze episodes were observed during the same winter period in 2014, each lasting approximately two days. The evolution of meteorological parameters and aerosol composition was classified into seven episodes shown in Fig. S2. Similar to the 2015 haze episode, the formation of these two episodes (F1 and F2, Fig. S2) was initiated by a change in air masses to the south, an increase of RH and a decrease of *T*. But, the evolution of the first haze episode (Ep2 and Ep3) was driven by southerly and southwesterly winds through the entire vertical layer (Fig. S2a). The ground wind speed was low (< 2 m s^{-1}) and higher wind speeds were observed at a higher altitude (> 6 m s^{-1} at 280 m). Under these conditions, the accumulation of secondary aerosol species was much slower than that in 2015. For example, it took nearly a day for sulfate and nitrate to reach approximately 20 μg m^{-3}, which was less than half the 2015 levels. The chemical composition of PM_1 during Ep2 was dominated by secondary aerosols, accounting for 58% on average. Sulfate, nitrate, and SOA were roughly equivalent contributing 13% to 15%. As indicated in Figure 3, the evolution of this episode was influenced by mountain-valley breezes at 00:00 on November 26 (M1 in Fig. S2) that substantially reduced secondary aerosol species. After the mountain-valley breezes disappeared and the wind direction switched back to southwesterly, aerosol species started to increase (Ep3) and the PM_1 mass concentration increased from 83 to 194 μg m^{-3}. Aerosol composition showed significant changes, with secondary aerosol species contributing 65% of PM_1 on average. In particular, sulfate increased and led to an enhancement in sulfate contribution from 14% to 20%. During this episode, variations in primary and secondary aerosol species were quite different. While the evolution of secondary aerosol species was principally driven by meteorological variations on a regional scale, primary aerosol species showed strong diurnal variations that appeared to be independent of meteorological conditions. Compared to the OA composition in the severe haze episode of 2015, POA was more significant than SOA in 2014, and accounted for 67% and 62% during Ep2 and Ep3, respectively. CCOA was the largest

POA component, on average contributing 26% and 34% of the total OA. This suggests that coal combustion emissions were the major source of OA during these two episodes.

The evolution of the second haze episode was similar to the first one. While the average mass concentration of PM_1 increased from 69 μg m^{-3} in the early formation stage (Ep5) to 129 μg m^{-3} during the fog stage (Ep6), sulfate increased the most from 16% to 24%. Secondary aerosol species dominated aerosol composition during both episodes, accounting for 72% and 67%, respectively. This highlights the dominant role of regional transport in the formation of severe haze episodes. But, we also noticed the considerable impact of local primary sources on OA, with contributions ranging from 53 – 66% during Ep2, Ep3, Ep5 and Ep6. Among POA components, CCOA was dominant, contributing 26 – 34% of OA. This further illustrates the important role of coal combustion emissions in the formation of winter haze.

Table S1. A summary of average aerosol species concentrations, optical properties, gaseous species, and meteorological parameters for the six episodes in Fig. 1.

	Ep1	Ep2	Ep3	Ep4	Ep5	Ep6
Species (μg m^{-3})						
SOA	2.32	24.50	27.46	27.12	41.19	0.99
SO_4	1.03	8.71	25.87	27.89	49.11	1.46
NO_3	0.62	13.17	22.22	26.13	31.89	0.18
NH_4	0.50	5.70	12.33	13.64	18.39	0.37
BC_{Sec}	0.32	4.08	8.93	10.00	14.69	0.30
POA	1.57	21.38	36.40	77.95	56.44	0.76
Chl	0.20	2.55	6.61	6.34	11.14	0.03
BC_{pri}	0.28	4.15	5.34	7.60	7.64	0.11
BC	0.72	7.18	13.53	16.49	23.54	0.33
PM_1	6.99	84.04	145.00	196.00	232.00	4.21
$PM_{2.5}$	4.10	131.30	267.00	334.30	462.30	3.70
Optical properties						
Ext (M m^{-1})	37.26	541.09	1270.11	1702.48	2693.21	26.71
Abs (M m^{-1})	5.25	52.45	98.76	120.40	171.88	2.43
SSA	0.86	0.90	0.92	0.93	0.94	0.91
Gas species (ppb)						
NO	4.79	9.74	76.84	249.71	140.54	3.63
NO_2	13.51	46.86	58.92	76.30	81.18	10.86
NO_x	18.30	56.60	135.76	326.02	221.72	14.49
CO	0.52	1.69	4.39	7.25	8.18	0.84
SO_2	3.19	6.57	23.31	14.75	13.85	23.41
O_3	27.64	3.27	0.74	0.88	0.38	14.85
Meteorological parameters						
RH, 8m (%)	35.6	53.4	65.4	73.8	85.0	31.6
RH, 100m (%)	34.1	55.1	70.3	59.0	97.4	30.9
T, 8m (°C)	-5.47	-3.34	0.10	3.01	2.11	3.74
T, 100m (°C)	-6.92	-4.64	-1.15	3.99	0.89	2.93
WS, 8m (m s^{-1})	2.65	1.17	1.02	0.72	0.90	2.90
WS, 100m(m s-1)	6.47	2.40	1.69	1.77	1.47	6.42

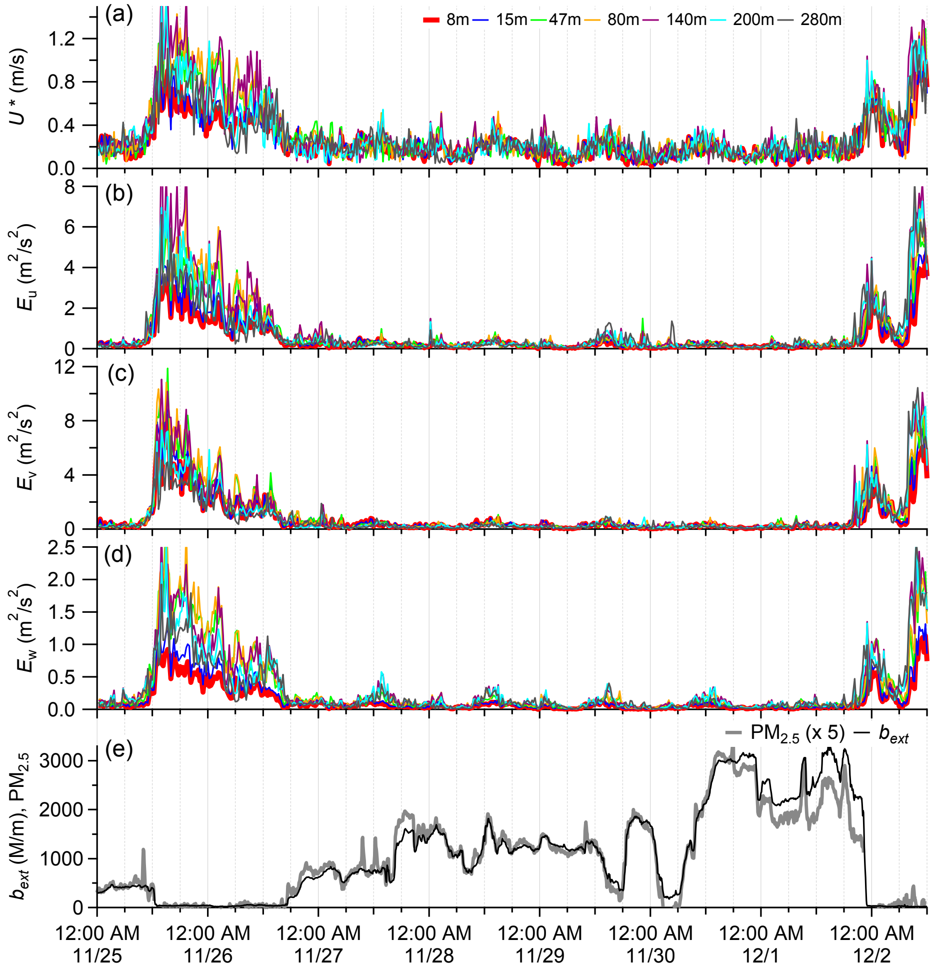

Figure S1. Time series of (a) friction velocity and (b-d) turbulence kinetic energy (TKE) in horizontal (*u* and *v*) and vertical (*w*) wind components at seven heights. (e) shows the time series of $PM_{2.5}$ mass concentration and particle extinction coefficient (b_{ext}). *u*, *v*, *w* are the three components of velocity along *x*, *y*, *z* directions, respectively.

The *u*, *v*, *w* are first averaged for 20 min for the "mean flows" ($\overline{u}, \overline{v}, \overline{w}$), and then the turbulence is calculated as the departure of the instantaneous wind from the 20 min mean, i.e., $u' = u - \overline{u}$. The turbulence kinetic energy of motion (*e*) along *x*, *y*, *z* directions in the period domain less than 20 min is calculated as $e_u = u'^2$, $e_v = v'^2$ and $e_w = w'^2$, respectively, and the averages of e_u, e_v and e_w for every 20 min (ensemble mean for one case) are denoted E_u, E_v and E_w, respectively, i.e., $E_u = \overline{u'^2}$, $E_v = \overline{v'^2}$, $E_w = \overline{w'^2}$. A more detailed description of the calculation of TKE is given in Cheng et al. [1]

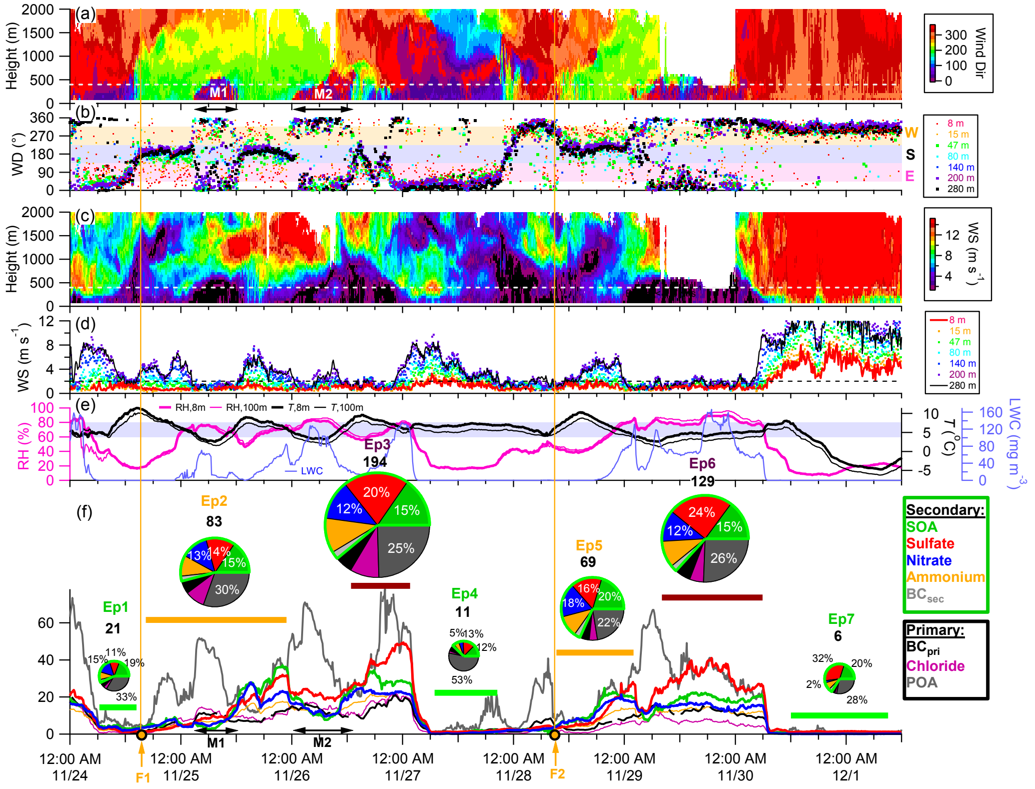

Figure S2. Evolution of (a, b) wind direction (WD), (c, d) wind speed (WS), (e) relative humidity (RH), temperature (*T*) and liquid water content (LWC), and (f) mass concentrations of aerosol chemical species in 2014. Seven episodes (Ep1 – Ep7) and two initial stages (F1 – F2) in the formation of the episodes are marked for further discussion. Pie charts show the average chemical composition of each episode. The horizontal dashed lines in (a) and (c) indicate a height of 400 m, and the dashed line in (d) indicates a wind speed of 2 m s^{-1}.

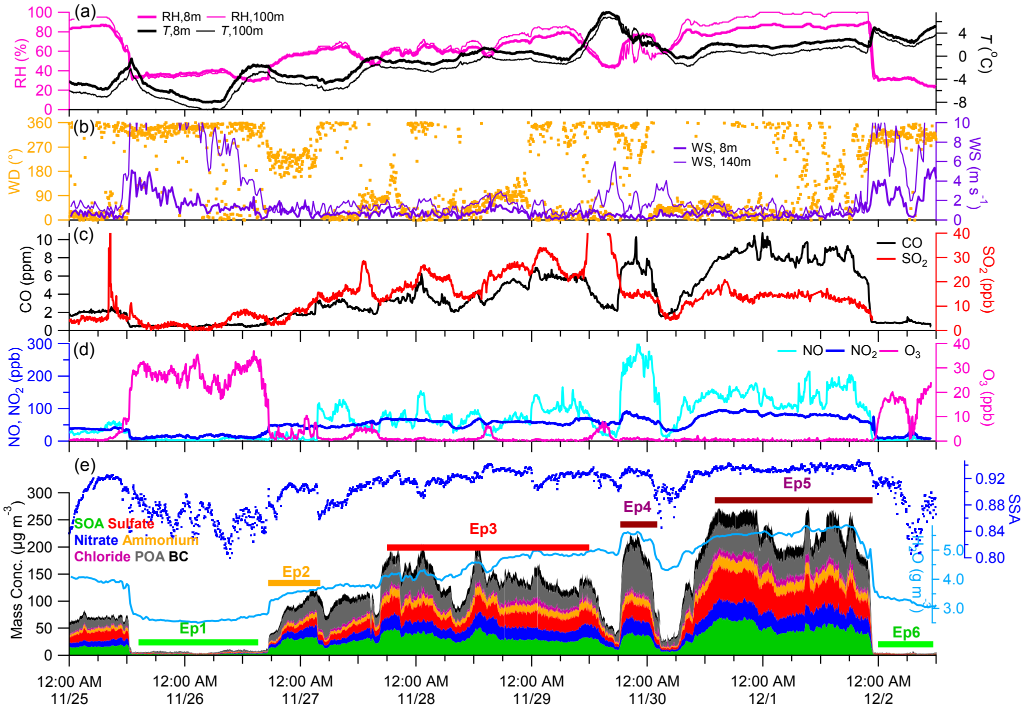

Figure S3. Time series of (a) relative humidity (RH) and temperature (T), (b) wind speed (WS) and wind direction (WD), (c) CO and SO_2, and (d) NO, NO_2, and O_3 from November 25 to December 2, 2015. (e) shows a stack plot of chemical composition of PM_1, single scattering albedo (SSA) and water vapor (H_2O) during the study period. Six episodes (Ep1 – Ep6) which are the same as in Figure 2 are marked.

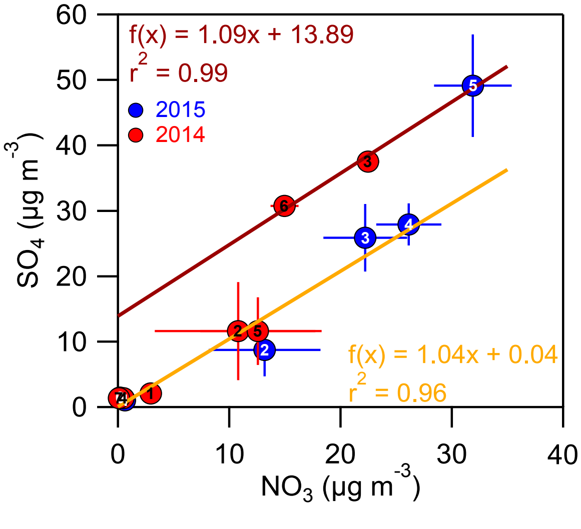

Figure S4. Scatter plot of sulfate versus nitrate for episodes (identified by numbers in the figure) in 2014 (red circles) and 2015 (blue circles). A linear fit was performed on the three episodes with significant aqueous-phase production of sulfate, and separately on the remaining episodes.

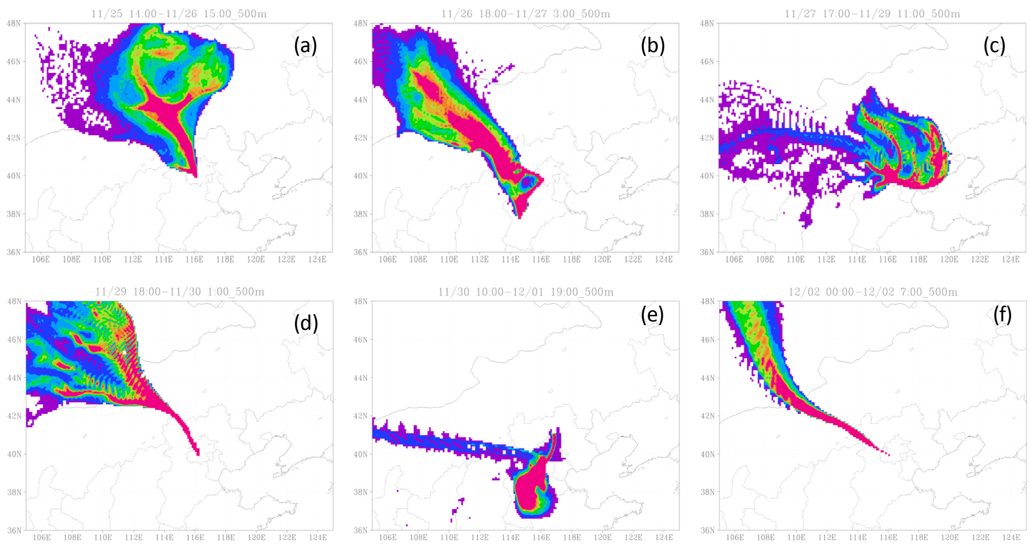

Figure S5. Footprint regions for air arriving at 500 m during the six episodes (Ep1 - Ep6) marked in Figures 1 and 2. The legend indicates the number concentrations of tracer particles. The maps were drawn by IGOR Pro (version 6.3.7.2, WaveMetrics, Inc., Oregon USA), http://www.wavemetrics.com/. The footprint region of each episode was determined using two-day backward simulations of the Lagrangian particle dispersion model FLEXPART [2] driven by the meteorological field (spatial resolution = 10 km, time resolution = 1 hour). In this study, the meteorological simulations were carried out using the Weather Research and Forecast model version 3.4 (WRF)[3] with the National Centers for Environmental Prediction (NCEP) global reanalysis data as the initial and boundary conditions. There were two domains in the simulation with grid resolutions of 30 and 10 km, respectively, and 28 vertical levels. In addition, the Yonsei University (YSU) boundary layer scheme, the Kain-Fritsch convective parametrization , the WSM3 microphysics scheme, the Dudhia shortwave scheme and the RRTM longwave scheme [4,5] were used in model simulations. Particle locations were then calculated with WRF-FLEXPART[6]. In the simulations, 10,000 tracer particles were released from the site at two heights, 50 m and 500 m, respectively, and the model was run backwards to determine the source areas and transport pathways of air pollutants during the specified period. A larger number of tracers in a cell indicated a greater impact from surface emission sources. A more detailed evaluation of WRF-FLEXPART is given elsewhere [7].

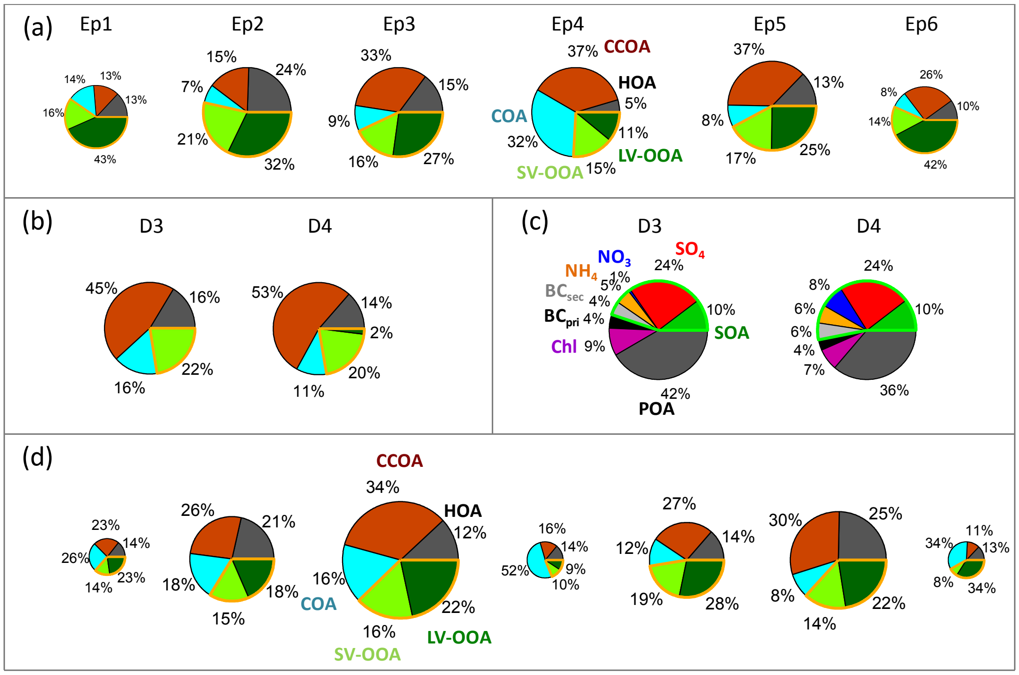

Figure S6. Average composition of organic aerosol during (a) six episodes (Ep1 – Ep6) and (b) two events (D3, D4) in 2015. (c) shows the average chemical composition of PM_1 for the D3 and D4 events in 2015, and (d) shows the average composition during seven episodes (Ep1 – Ep7) in 2014. The episode information in 2015 and 2014 is marked in Figure 2 and Figure 3, respectively.

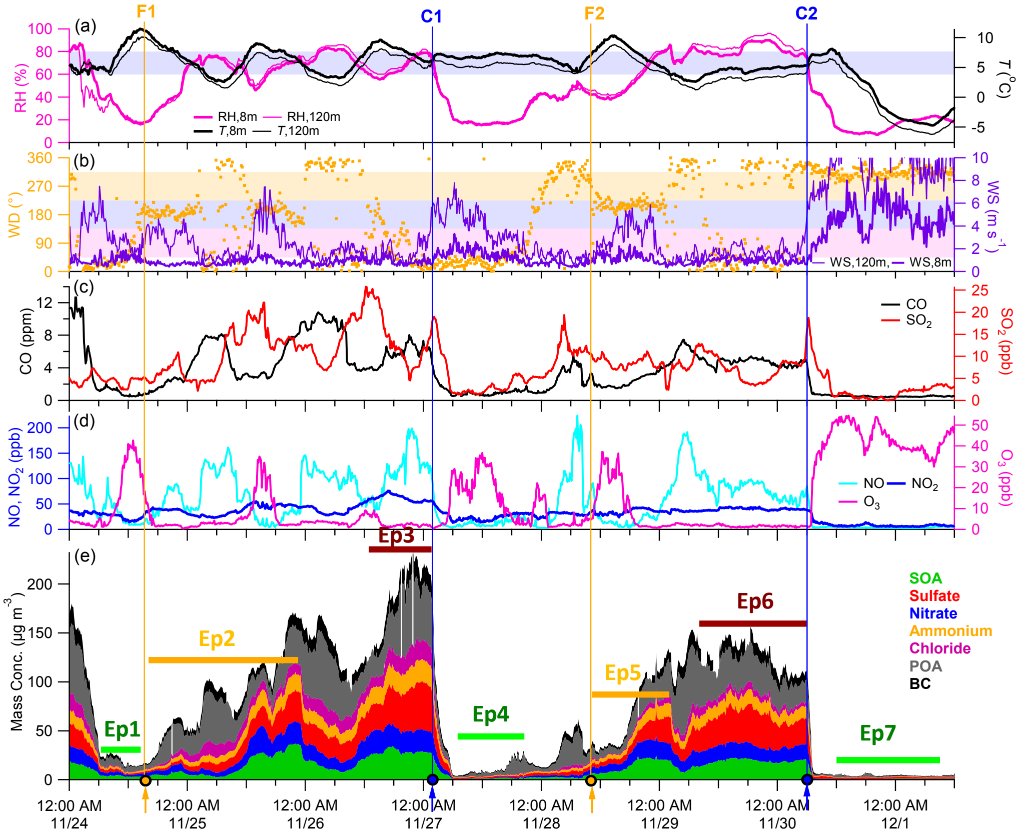

Figure S7. Time series of (a) relative humidity (RH) and temperature (*T*), (b) wind speed (WS) and wind direction (WD), (c) CO and SO_2, and (d) NO, NO_2, and O_3 from November 24 to December 1, 2014. (e) shows a stack plot of chemical composition of PM_1 during the study period. Seven episodes (Ep1 – Ep7) are identified and are included in the analysis for Figure 3.

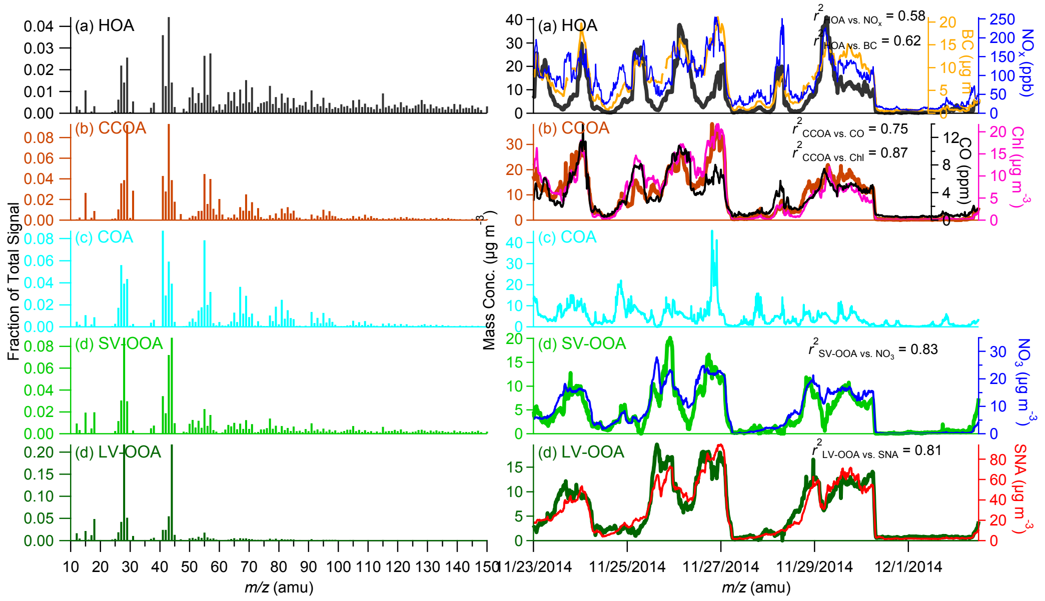

Figure S8. Mass spectra (left panel) and time series (right panel) of five OA factors resolved from PMF analysis of organic aerosol spectra in 2014. A comparison of OA factors with the external tracers is also shown in the right panel.

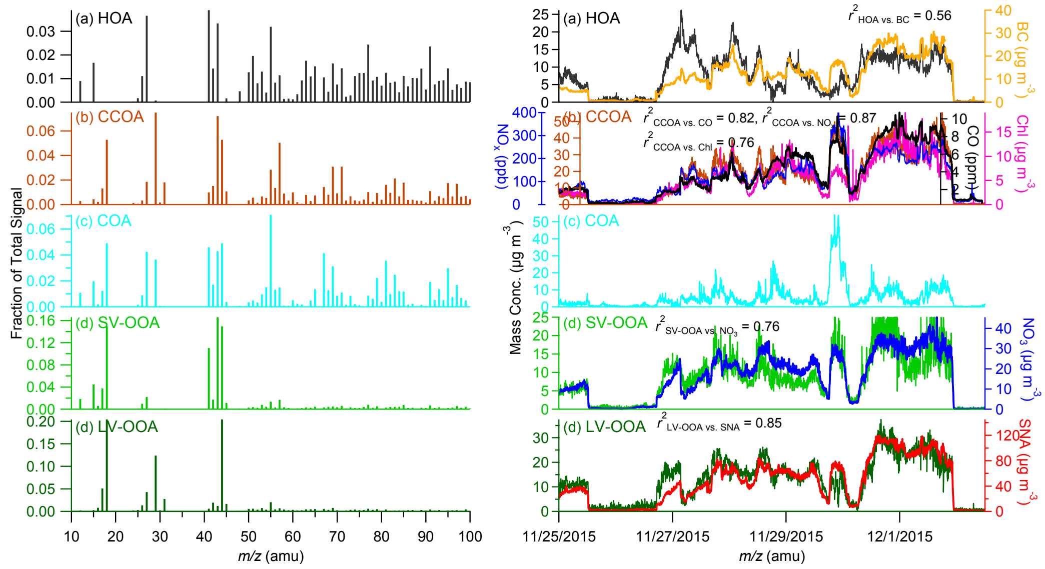

Figure S9. Mass spectra (left panel) and time series (right panel) of five OA factors resolved from PMF analysis of organic aerosol spectra in 2015. A comparison of OA factors with the external tracers is also shown in the right panel.

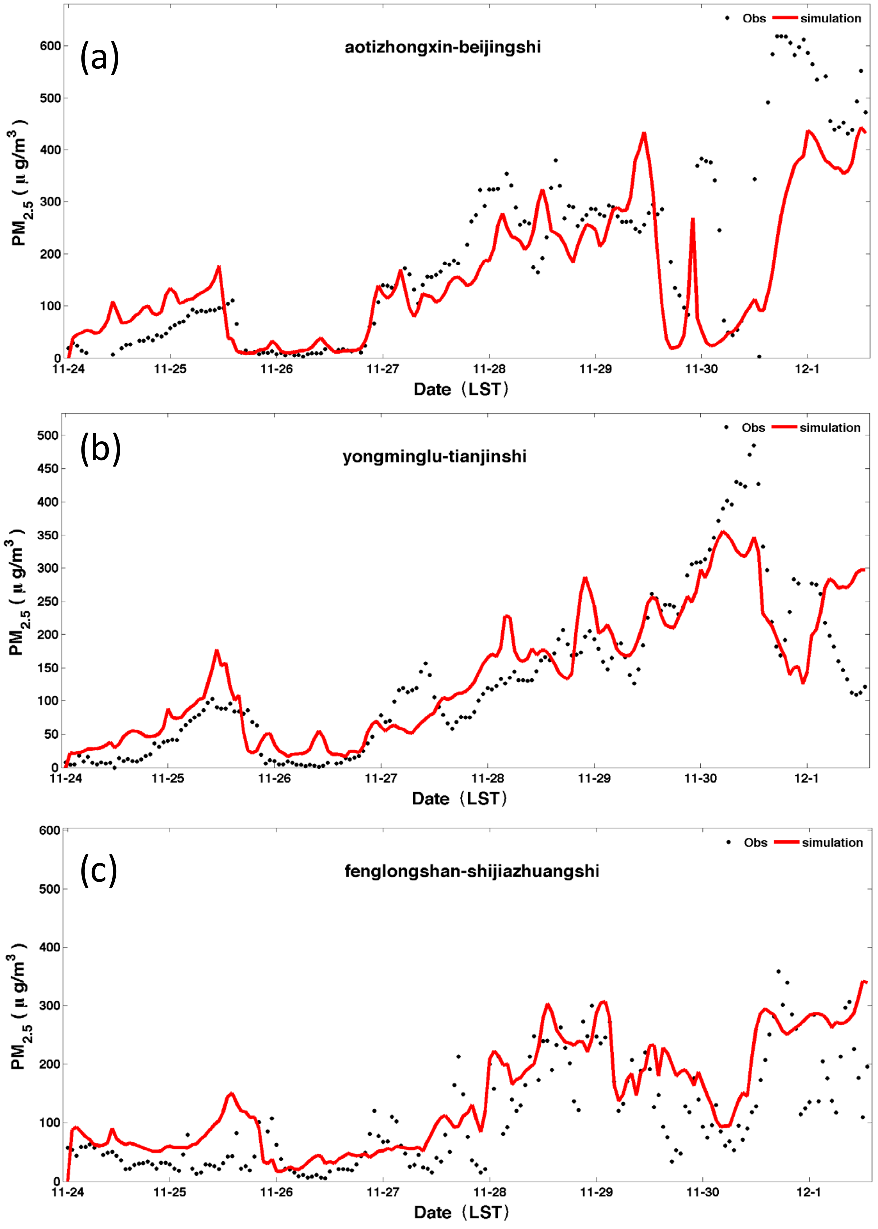

Figure S10. Comparisons of the simulated and observed $PM_{2.5}$ concentrations from November 24, 2015 to December 1, 2015 at (a) Aoti centre in Beijing, (b) Yongminglu in Tianjing, and (c) Fenglongshan in Shijiazhuang.

References:

1 Cheng, X., Zeng, Q. C. & Hu, F. Characteristics of gusty wind disturbances and turbulent fluctuations in windy atmospheric boundary layer behind cold fronts. *Journal of Geophysical Research: Atmospheres* **116** (2011).

2 Stohl, A., Forster, C., Frank, A., Seibert, P. & Wotawa, G. Technical note: The Lagrangian particle dispersion model FLEXPART version 6.2. *Atmospheric Chemistry and Physics* **5**, 2461-2474 (2005).

3 Skamarock, W. C. *et al.* A description of the advanced research WRF version 2. (DTIC Document, 2005).

4 Hong, S.-Y., Noh, Y. & Dudhia, J. A new vertical diffusion package with an explicit treatment of entrainment processes. *Mon Weather Rev* **134**, 2318-2341 (2006).

5 Kain, J. S. The Kain-Fritsch convective parameterization: an update. *J Appl Meteorol* **43**, 170-181 (2004).

6 Fast, J. D. & Easter, R. C. in *7th WRF User's Workshop, Boulder, CO, USA.* (Citeseer).

7 de Foy, B. *et al.* Aerosol plume transport and transformation in high spectral resolution lidar measurements and WRF-Flexpart simulations during the MILAGRO Field Campaign. *Atmos. Chem. Phys.* **11**, 3543-3563, doi:10.5194/acp-11-3543-2011 (2011).
